# Restoration of SMN in Schwann cells reverses myelination defects and improves neuromuscular function in spinal muscular atrophy

**DOI:** 10.1093/hmg/ddw141

**Published:** 2016-05-11

**Authors:** Gillian Hunter, Rachael A. Powis, Ross A. Jones, Ewout J.N. Groen, Hannah K. Shorrock, Fiona M. Lane, Yinan Zheng, Diane L. Sherman, Peter J. Brophy, Thomas H. Gillingwater

**Affiliations:** 1 Department of Life Sciences, School of Health and Life Sciences, Glasgow Caledonian University, Glasgow G4 0BA, UK,; 2 Euan MacDonald Centre for Motor Neurone Disease Research, University of Edinburgh, Edinburgh, EH16 4SB, UK,; 3 Centre for Integrative Physiology, University of Edinburgh, Edinburgh, EH8 9XD, UK and; 4 Centre for Neuroregeneration, University of Edinburgh, Edinburgh, EH16 4SB, UK

## Abstract

Spinal muscular atrophy (SMA) is a neuromuscular disease caused by low levels of SMN protein, primarily affecting lower motor neurons. Recent evidence from SMA and related conditions suggests that glial cells can influence disease severity. Here, we investigated the role of glial cells in the peripheral nervous system by creating SMA mice selectively overexpressing SMN in myelinating Schwann cells (*Smn*^−/−^;*SMN2*^tg/0^;*SMN1*^SC^). Restoration of SMN protein levels restricted solely to Schwann cells reversed myelination defects, significantly improved neuromuscular function and ameliorated neuromuscular junction pathology in SMA mice. However, restoration of SMN in Schwann cells had no impact on motor neuron soma loss from the spinal cord or ongoing systemic and peripheral pathology. This study provides evidence for a defined, intrinsic contribution of glial cells to SMA disease pathogenesis and suggests that therapies designed to include Schwann cells in their target tissues are likely to be required in order to rescue myelination defects and associated disease symptoms.

## Introduction

Proximal spinal muscular atrophy (SMA) is an autosomal recessive neuromuscular condition, with an incidence of ∼1 in 6000–11 000 live births (1,2). The major pathological characteristic of SMA is a loss of lower alpha motor neurons from the ventral horn of the spinal cord, resulting in progressive muscle atrophy and eventual paralysis. SMA is primarily caused by homozygous deletion of, or mutations in, the *survival of motor neuron 1* (*SMN1*) gene (3). Its full-length protein product, SMN, is a ubiquitous and essential cellular protein. In humans, there are two *SMN* genes, *SMN1* and *SMN2* (4)*.* However, a base pair substitution in *SMN2* results in increased exclusion of Exon 7 from transcripts and production of an unstable transcript (5,6). *SMN2* therefore produces considerably less full-length SMN protein than *SMN1* and cannot fully compensate for its loss (4).

Motor neurons are particularly vulnerable to low levels of SMN (4). However, with the emergence of SMA animal models and a clearer appreciation of disease pathology in human patients, it has become apparent that reduced levels of SMN have additional effects on multiple other cell-types and tissues (7). For example, skeletal muscle cells in cultures from severe SMA patients show defective development and are disorganized (8,9); cardiac arrhythmias and abnormalities are one of the most common peripheral phenotypes reported in patients with severe SMA (10); while vascular (11–15), liver (16,17), metabolic (18) and consistent intestinal and lung (13) abnormalities have also been reported.

In addition, there is a growing evidence from studies of related motor neuron diseases, such as amyotrophic lateral sclerosis (ALS), that non-neuronal cells can play a defining role in regulating motor neuron degeneration (19). This may have particular relevance for SMA in light of recent studies showing that restoration of SMN in motor neurons had only modest effects on the SMA phenotype (20–23).

An evidence of gliosis in SMA patients has been known for some time (24–26) and recent work has suggested that astrocytes in particular may contribute to SMA pathogenesis in the central nervous system (CNS). For example, morphological and cellular alterations in astrocytes occur prior to the loss of motor neurons (27). Similar evidence for astrogliosis has been presented from post-mortem patient spinal cords (28). Glial cells from the peripheral nervous system (PNS) have also been implicated in SMA, with changes observed in non-myelinating terminal Schwann cells in several different SMA mouse models (29–31). Similarly, *in vivo* and *in vitro* analyses of myelinating Schwann cells demonstrated intrinsic defects in SMA (evidenced by SMN-dependent failure of isolated SMA-derived Schwann cells to respond to myelination cues), leading to defects in myelination and generation of extracellular matrix in peripheral nerve (32,33).

Despite accumulating evidence for intrinsic pathology of Schwann cells in SMA, it remains unknown whether Schwann cells directly influence motor neuron pathology. Answering this question will be important, not only to better understand the contribution of cell autonomous and non-cell autonomous pathways to motor neuron degeneration in SMA, but also to identify critical targets for delivering effective therapies for the treatment of SMA.

## Results

### Generation of SMN1^SC^ transgenic mice

**Figure 1.**
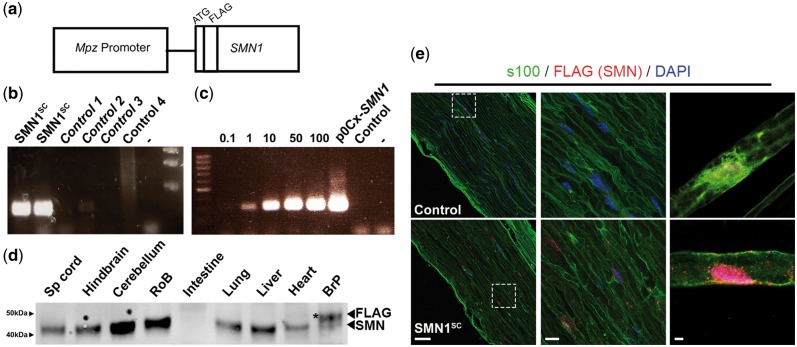
Restoration of SMN in myelinating Schwann cells. (**A**) A p*Mpz*-*SMN*, construct was generated by inserting the *SMN1* gene downstream of the myelin protein zero (*Mpz*) promoter, incorporating a 5′ FLAG tag. (**B**) Oligonucleotides were designed to uniquely amplify the *SMN1* gene demonstrated by amplification of *SMN1*^SC^ but not of endogenous mouse *Smn1* [control 1 (*Smn*^+/−^, FVB), control 2 (wild-type, FVB), control 3 (wild-type, CD1)] or human *SMN2* [Control 4 (*Smn*^−/−^*:SMN2^tg/tg^*)]. Negative sample (−). (**C**) The amplification reaction had high sensitivity to the *SMN1* transgene and was able to detect at least 1 copy of the p*Mpz*-*SMN1* transgene [0.1, 0.1 copy p0Cx-*SMN1* (0.298 ρg p0Cx-*SMN1*); 1, 1 copy p0Cx-*SMN1* (2.98 ρg p0Cx-*SMN1*); 10, 10 copies p0Cx-*SMN1* (29.8 ρg p0Cx-*SMN1*); 50, 50 copies p0Cx-*SMN1* (149 ρg p0Cx-*SMN1*); 100, 100 copies p0Cx-*SMN1* (298 ρg p0Cx-*SMN1*)], control (wild-type, CD1), negative sample (−); 5 µl total PCR product loaded per well. (**D**) Representative immunoblot of tissue from *Smn^+/+^*;*SMN1*^SC^ mice demonstrating selective FLAG-*SMN1* expression in peripheral nerve (sciatic nerve), but not in the CNS or systemic tissue. Asterisk (*) indicates FLAG-tagged SMN expressed from the p*Mpz-SMN* construct; 25 µg protein loaded per well. (**E**) Sectioned (left and middle panels) and teased sciatic nerve fibres (right panels) confirming the expression of FLAG-SMN in PNS of *Smn^+/+^*;*SMN1*^SC^ mice (SMN1^SC^), but not in *Smn^+/+ ^*mice (control, FVB). Scale bars = 20 μm (left and middle panels, E), 10 μm (right panels, E).

To determine the effect of restoring SMN selectively in myelinating Schwann cells, we generated a novel transgenic mouse line where human *SMN1* cDNA was placed under the control of a hybrid *Mpz/P0-Cx32* construct known to drive efficient expression of transgenes in myelinating Schwann cells (34) (Fig. 1A). *Mpz*-driven constructs do not express in motor neurons, CNS glia or non-myelinating PNS glia (e.g. terminal Schwann cells) (34). *Mpz/P0-Cx32*-driven *SMN1* constructs were microinjected into fertilized mouse oocyte to generate wild-type transgenic mice expressing human *SMN1* in myelinating Schwann cells (*SMN1*^SC^). To identify mice carrying the human *SMN1* gene we developed a specific and sensitive qualitiative PCR assay that exclusively amplified *SMN1* (Fig. 1B and C). The primers did not amplify murine S*mn* (Controls 1–3; Fig. 1B) or the human *SMN2* transgene (Control 4; Fig. 1B) and were capable of detecting at least one copy of human *SMN1* (Fig. 1C). Two *SMN1*^SC^ transgene-positive founders were generated (GO30 and GO40) from a total of 68 live pups. A backcross of the GO40 founder mouse with an FVB/N mouse yielded 83% transgene positive pups. A similar cross between the GO30 founder mouse and an FVB/N mouse generated no transgene positive pups.

FLAG-SMN (1 kDa larger than SMN) was expressed in peripheral nerve, but not in a range of other tissues (muscle, brain, heart, liver and spinal cord) in the GO40 line, indicating that FLAG-SMN expression was indeed limited to the PNS, as expected (Fig. 1D). PNS expression was confirmed by immunohistochemical analyses of sectioned and teased nerve fibres from sciatic nerve; an antibody to the 5′ FLAG tag on the transgene demonstrated clear expression of FLAG-SMN in sciatic nerve co-stained with S100, a Schwann cell marker (Fig. 1E).

### *Over-expression of* SMN *in myelinating Schwann cells does not affect normal neuromuscular development*

**Figure 2.**
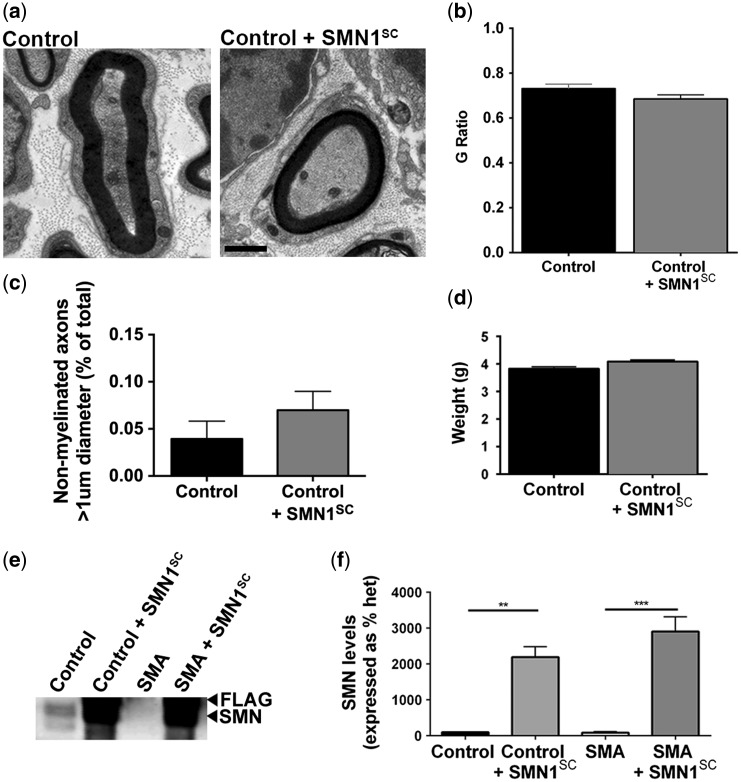
Overexpression of *SMN1* in Schwann cells is not detrimental. (**A**) Representative electron micrograph of a large diameter myelinated axon from the intercostal nerve of a mouse expressing *SMN1*^SC^. (**B**) Intercostal nerves from control mice expressing SMN^SC^ had no difference in *G*-ratio measurements compared with control mice (two-tailed, unpaired *t-*test; *N* = 3 mice per genotype, *n *> 50 independent nerve fibre measurements per genotype). (**C**) No significant increase in numbers of unmyelinated large diameter (>1 μm) axons at P7 in control mice expressing *SMN1*^SC^ (*N* = 3) (**D**) Restoration of SMN to Schwann cells had no detrimental effect on the weight of mice (*N* > 35 mice per genotype). All tests two-tailed, unpaired *t-*tests; *N* = 3 mice per genotype, *n *> 50 independent nerve fibre measurements per genotype. Scale bar = 1 μm (A). (**E**) Representative immunoblot of intercostal nerve tissue from control, transgenic control mice (control + *SMN1*^SC^), ‘Taiwanese” SMA mice (SMA) and SMA rescue mice (SMA + *SMN1*^SC^) demonstrating selective SMN1 overexpression in mice carrying the p*Mpz*-*SMN* construct. (**F**) SMN1 levels were normalized using tubulin levels as a loading control and demonstrated statistically significant SMN1 overexpression in mice carrying the p*Mpz*-*SMN* construct.

Next, we wanted to confirm that overexpression of SMN in myelinating Schwann cells was not detrimental to normal neuromuscular development. At the gross anatomical level, mice expressing *SMN1*^SC^ were indistinguishable from control animals, with similar survival (Fig. 4H) and breeding rates (data not shown). We confirmed that SMN was significantly overexpressed in *SMN1*^SC^ positive control mice (control + *SMN1*^SC^) compared with wild-type mice (control) (∼2000-fold) (Fig. 2E and F). To investigate potential effects at the cellular level, we carried out an assessment of intercostal nerves from P7 mice expressing the *SMN1* transgene. Measurement of axon thickness and axon plus myelin thickness allows a *G*-ratio to be calculated. *G*-Ratio measurements enable assessment of myelin thickness with increased *G*-ratios indicating a reduction in average myelin sheath thickness relative to the axon diameter. There was no qualitative or quantitative difference in myelin sheath thickness in mice expressing *SMN1*^SC^ (Fig. 2A and B). There was also no change in the number of non-myelinated large diameter (>1 µm) axons (Fig. 2C) or animal weight (Fig. 2D). Thus, supra-physiological levels of SMN protein are well tolerated by Schwann cells, with no overt impact on normal peripheral nerve myelination.

Following confirmation that the GO40 line expressed SMN as expected and that there were no detrimental effects because of overexpression of SMN in myelinating Schwann cells, we backcrossed these mice onto a FVB/N background until 98.4% homozygosity was reached. Experimental ‘rescue’ animals (mice lacking endogeneous *Smn1*, heterozygous for *SMN2* and expressing *SMN1* in Schwann cells; *Smn*^−/−^;*SMN2*^tg/0^;*SMN1*^SC^ or SMA + *SMN1*^SC^) were then generated by crossing *SMN1*^SC^ mice with the severe ‘Taiwanese’ SMA line (*Smn*^−/−^;*SMN2*^tg/tg^ or SMA) (34−36). We confirmed that SMN was significantly overexpressed in *SMN1*^SC^ positive mice (∼2000-fold in control + *SMN1*^SC^; ∼3000-fold in SMA + *SMN1*^SC^ mice compared with non-*SMN1*^SC^ carrying control and SMA mice (Fig. 2E and F).

### *Restoration of* SMN *to myelinating Schwann cells rescues myelin sheath defects in SMA mice*

Previously, we reported that intercostal nerves from Taiwanese SMA mice had defective myelination, manifesting as a thinner myelin sheath and a larger number of large diameter unmyelinated axons at both mid- (P7) and late- (P11) symptomatic time points (33). To evaluate whether restoration of SMN expression in myelinating Schwann cells was sufficient to ameliorate these myelin defects we assessed intercostal nerve myelination in experimental rescue mice (SMA + *SMN1*^SC^) at a mid-/late-symptomatic time point (P7). Intercostal nerves were selected for these experiments because of the findings from previous results showing that the most robust myelination defects observed in Taiwanese SMA are to be found in intercostal nerves (e.g. compared with sciatic nerve) (33).

**Figure 3.**
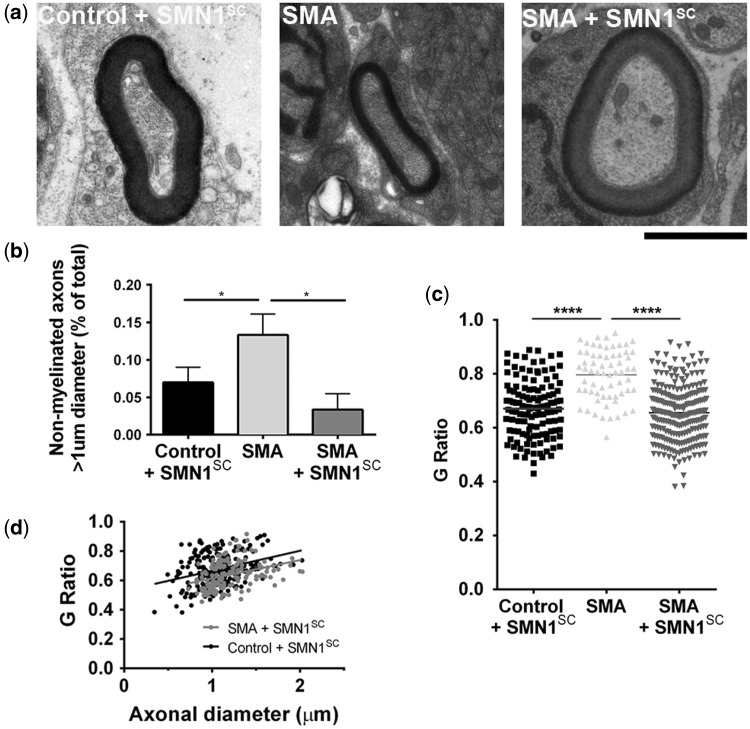
Restoring *SMN* expression in Schwann cells rescues myelination defects in SMA mice. (**A**) Representative electron micrographs of large diameter (>1 µm) myelinated axons in intercostal nerves from littermate control mice (control + *SMN1*^SC^), ‘Taiwanese” SMA mice (SMA), and SMA rescue mice (SMA + *SMN1*^SC^) at P7. (**B**) A significant rescue in numbers of unmyelinated large diameter (> 1 μm) axons in SMA rescue mice at P7. (**C**) A significantly lower average *G*-ratio in intercostal nerves from rescue SMA mice at P7 compared with ‘Taiwanese’ SMA mice (SMA), with higher *G*-ratios across the range of axon calibers (**D**). *P < 0.05, ****P < 0.0001; all tests two-tailed, unpaired *t-*tests; *N* = 3 mice per genotype, *n *> 50 independent nerve fibre measurements per genotype. Scale bar = 1.5 μm (A).

Qualitatively, myelin thickness in SMA rescue mice appeared closer to that observed in control littermates than SMA mice (Fig. 3A). Quantitative analyses revealed a significant rescue of the myelin sheath pathology in SMA rescue mice with a significantly reduced average *G*-ratio (0.656 ± 0.007) compared with SMA mice (0.796 ± 0.012), restored to levels observed in controls (0.670 ± 0.009) (Fig. 3C). Restoration of myelin thickness was observed across a full range of axonal calibres, with no evidence for reduced axon diameters in the SMA rescue mice (Fig. 3D). In addition, there was a significant reduction in the number of non-myelinated large diameter (>1 µm) axons in SMA rescue mice (Fig. 3B).

Taken together, these findings support the hypothesis that myelination defects in SMA result from direct, intrinsic deficiencies in Schwann cells, rather than occurring as a secondary consequence to pathology in neighbouring motor neurons (32,33).

### *Restoration of* SMN *to myelinating Schwann cells improves neuromuscular function and ameliorates neuromuscular junction pathology without affecting motor neuron soma loss or systemic pathology*

**Figure 4.**
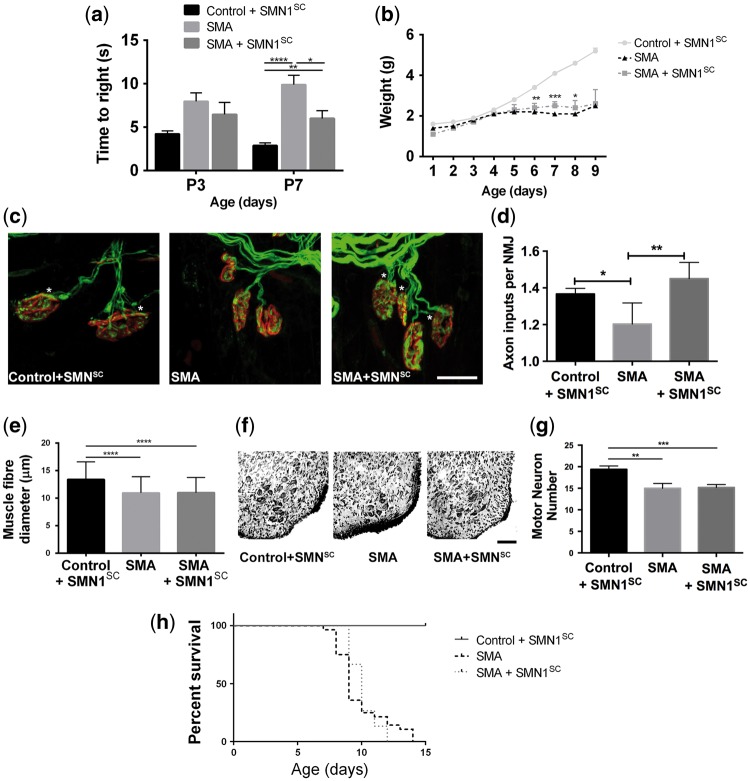
Restoring *SMN* expression in Schwann cells improves neuromuscular function and reverses NMJ pathology in SMA mice. (**A**) Significant improvement in time to right in mid/late-symptomatic (P7) SMA rescue mice (SMA + *SMN1*^SC^) compared with SMA littermates (P3, *N* = 18 mice control + *SMN1*^SC^; *N* = 35 SMA; *N* = 19 SMA + *SMN1*^SC^; P7, *N* = 32 mice control + *SMN1*^SC^; *N* = 60 SMA; *N* = 33 SMA + *SMN1*^SC^) (two-tailed, unpaired *t-*test). (**B**) Modest, but significant improvement in body weight in SMA rescue mice (*N* = 3 − 64 mice per genotype/time point; two-tailed, unpaired *t-*test). (**C** and **D**) Reduced NMJ pathology (measured as the average number of axonal inputs per NMJ; multiply innervated NMJs are indicated by asterisks) in the LAL muscle of SMA rescue mice (SMA + *SMN1*^SC^*)* compared with SMA littermates at P8/9 (*N* = 3 mice, control + *SMN1*^SC^; *N* = 7 mice, SMA; *N* = 4 mice, SMA + *SMN1*^SC^*; n *> 50 NMJs per muscle) (two-tailed, unpaired *t-*test). (**E**) No amelioration of skeletal muscle fibre atrophy in the LAL muscle of SMA rescue mice at P8/9 compared with SMA littermates (*N* = 3 mice control + *SMN1*^SC^ and SMA; *N* = 4 SMA + *SMN1*^SC^; *n* = 50 measurements; two-tailed, unpaired *t-*test). (**F** and **G**) No amelioration of motor neuron loss from the ventral horn of spinal cord in SMA rescue mice at P8/9 compared with SMA littermates (*N* = 3 mice control + *SMN1*^SC^ and SMA; *N* = 4 SMA + *SMN1*^SC^; *n* = 4; two-tailed, unpaired *t-*test). (**H**) No significant difference in survival of SMA rescue mice (*N* = 25 mice control + *SMN1*^SC^; *N* = 28 SMA; *N* = 15 SMA + *SMN1*^SC^) (Mantel−Cox test). *P < 0.05, **P < 0.01, ***P < 0.001, ****P < 0.0001. Scale bars = 20 μm (C) and 100 µm (F).

We next wanted to establish whether restoring SMN levels in Schwann cells could have any impact on neuromuscular function and/or motor neuron pathology. Initial tests of neuromuscular function [using an established ‘righting test’ (37)] revealed that SMA rescue mice (SMA + *SMN1*^SC^) had a clear functional improvement at a mid-/late-symptomatic time-point (P7) compared with SMA mice (Fig. 4A). Although not maintained through to the end-stage of disease, SMA rescue mice also had modest, but significant, improvements in weight at P6, P7 and P8 (Fig. 4B).

Neuromuscular junction (NMJ) pathology is a major hallmark of motor neuron degeneration in SMA, with denervation and delayed motor endplate maturation being observed in many different mouse models of severe SMA (38,39). However, in our hands, the only robust NMJ pathology observed in the ‘Taiwanese’ mouse model of SMA is a reduced number of axonal inputs to NMJs, particularly notable in the *levator auris longus* (LAL) muscle (37). We therefore wanted to establish whether the improvements in neuromuscular function we observed in SMA rescue mice (SMA + *SMN1*^SC^) correlated with an amelioration of this aspect of NMJ pathology. We found a significant increase in the number of axonal inputs to NMJs in the *LAL* of SMA rescue mice, with the average number of inputs restored to levels observed in controls (P8/9; Fig. 4C and D). Interestingly, these improvements in NMJ pathology occurred in the absence of any parallel improvement in skeletal muscle, with no rescue in the smaller muscle fibre diameters (from the *LAL* muscle) in the SMA rescue mice compared with SMA mice (P8/9; Fig. 4E).

Improvements in neuromuscular function and NMJ pathology observed in SMA rescue mice (SMA + *SMN1*^SC^) occurred in the absence of any significant improvement in levels of motor neuron soma loss from the ventral horn of spinal cord (Fig. 4F and G). The number of motor neurons in the ventral horn of spinal cord was reduced by ∼25% in SMA mice compared with controls, as previously reported (40). However, similar reduced numbers of motor neurons were also observed in the SMA rescue mice (SMA + *SMN1*^SC^).

Previous reports have suggested that restoration of SMN solely in motor neurons improves motor neuron pathology, but only modestly influences systemic SMA pathology (20–23). We therefore wanted to establish whether restoring SMN in myelinating Schwann cells, with concurrent improvements in myelination, neuromuscular function and NMJ pathology, resulted in any improvements in systemic pathology. Restoration of SMN in myelinating Schwann cells did not lead to any increase in survival or improvements in the gross appearance of SMA mice (Fig. 4H, data not shown), indicating selective effects of targeting Schwann cells on glial/motor neuron pathology and resulting neuromuscular function.

## Discussion

In this study we have demonstrated that selective restoration of SMN in Schwann cells can lead to marked improvements in selective aspects of disease pathogenesis in a mouse model of severe SMA. Elevated levels of SMN in Schwann cells were well tolerated and did not influence normal Schwann cell or neuromuscular development. Restoring SMN in Schwann cells was sufficient to reverse the myelination defects that occur in SMA, suggesting that these occur because of an intrinsic defect in the glia cells, rather than as a secondary consequence of degenerative events occurring in neighbouring motor neurons. Alongside improvements in myelination, elevated levels of SMN in Schwann cells improved neuromuscular function and aspects of NMJ pathology. This indicates that at least some of the pathological changes and functional defects occurring in the neuromuscular system during severe forms of SMA are a consequence of defective glial cells. Whether such defects are also occurring in less severe forms of the disease needs to be established, and should be aided by the recent development of milder mouse models of SMA (e.g. *SMN2B/−* mice).

One interesting observation made during the current study was that neuromuscular function and NMJ pathology were ameliorated in the absence of overt improvements in motor neuron loss or skeletal muscle fibre atrophy. This suggests that improvements in some aspects of neuromuscular pathology in SMA can be generated by interventions that do not directly target motor neurons and/or skeletal muscle. Thus, improving the health of myelinating Schwann cells alone would be predicted to have a significant impact on NMJ stability of neighbouring motor neurons, and also improve neuromuscular function in SMA patients (41). The precise mechanisms through which Schwann cells can influence morphological and functional aspects of neuromuscular connectivity in health and disease are only beginning to be uncovered. However, our findings in SMA are consistent with the recent demonstration that robust Schwann cell–motor neuron interactions are required in order to sculpt appropriate synaptic connectivity at the NMJ during development in neonatal mice (42).

Our findings provide significant additional experimental support for a model whereby SMA represents a complex, multi-factorial disease, where disease symptoms cannot simply be ascribed to changes occurring solely in motor neurons and skeletal muscle fibres (7,43). Thus, therapeutic targeting of motor neurons alone is unlikely to be sufficient to ameliorate all aspects of SMA disease pathogenesis. For example, we previously reported significant amelioration of the neuromuscular phenotype, with no apparent influence on systemic pathology, in SMA mice following delivery of a β-catenin inhibitor (37). Moreover, viral gene therapy approaches used to restore SMN to astrocytes in the Δ7 model of SMA reported significant improvement in motor neuron-associated pathology, but again only produced a modest amelioration of systemic pathology and lifespan (28). These findings are also in agreement with several studies that together suggest that restoration of SMN solely within motor neurons (returning SMN levels to those seen in wild-type healthy controls) leads to unexpectedly minor phenotypic improvements (20–23). Thus, therapies designed to restore SMN levels in human SMA patients would likely benefit from the ability to target Schwann cells (and other similar cell types) alongside motor neurons, if glial cell-associated defects are also to be successfully ameliorated.

In conclusion, whilst there is currently no cure for SMA, there is increasing evidence from pre-clinical trials that viral gene therapy approaches, and antisense oligonucleotide approaches can be both efficacious and translationally viable with respect to restoring SMN levels *in vivo* (44). Our findings indicate that therapies capable of targeting SMN-dependent pathology in glial cells, alongside other critical disease targets, such as motor neurons and skeletal muscle, are likely to be required in order to ameliorate the full range of neuromuscular pathology observed in SMA.

## Materials and Methods

### pMPZ-SMN construct generation

The p*Mpz*-*SMN1* transgene was created using a two-step cloning procedure from two starting plasmids: plasmid containing the full-length human *SMN1* construct and the hybrid *Mpz/P0-Cx32* construct that contains a 1.1 kb fragment containing the rat *Mpz* promoter fused to exons 1b and 2 and the intervening intron from the human *Cx32* gene (34) (Fig. 1A). Briefly, *SMN1* was amplified using two overlapping forward primers that introduced *Nhe*I and *Asc*I sites, an ATG start site and a 5′ FLAG tag (F1, 5′–GCTAGCTAGCAGGCGCGCCATGGATTACAAGGATGACGACGATAAG-3′; F2, 5′–AAGGATGACGACGATAAGGGAGGTGCGATGAGCAGCGGCGGC-3′) and a reverse primer that introduced *Bgl*II and *Aat*II sites and a TGA stop site (R, 5′-GGCTAAGATCTTGACGTCAATTTAAGGAATGTGAGCACCTTCC-3′). Amplifed products were digested using *Nhe*I and *Bgl*II (New England Biolabs), ligated to an intermediate plasmid (pGL4.13, Promega) using T4 DNA ligase (Promega), then confirmed by sequencing. The intermediate *SMN1*-containing plasmid and the *Mpz*-promoter plasmid were digested using *Asc*I and *Aat*II (New England Biolabs) and ligated using T4 DNA ligase. p*Mpz*-*SMN1* constructs were confirmed by restriction digestion and sequencing.

### Pronuclear injection

Fifty micrograms of p*Mpz*-*SMN1* were digested with *MluI* and *NotI* (New England Biolabs), to release the 5.8 kb transgene cassette. Transgenic injections were performed as previously described (45).

### Genotyping of SMN1^SC^ transgene

A PCR capable of detecting human *SMN1* was optimized using a forward primer located in *SMN1* Exon 3 (5′-ACCACACCTAAAAGAAAACCTGCT-3′) and a reverse primer that spanned *SMN1* Exon 4 to Exon 5 (5′-TTTCATTTTCATTCTCTTGAGCA-3′).

### Spiked PCR assay/transgene PCR standard

To determine the sensitivity of the transgene genotyping reaction 200 ng genomic DNA (CD1 mice tail tip) was spiked with DNA from a plasmid expressing *SMN1*, p0Cx-*SMN1*, to generate a series of copy number standards; 0.1 copy (0.298 ρg p0Cx-*SMN1*); 1 copy (2.98 ρg p0Cx-*SMN1*); 10 copies (29.8 ρg p0Cx-*SMN1*); 50 copies (149 ρg p0Cx-*SMN1*); 100 copies (298 ρg p0Cx-*SMN1*). Amounts of DNA used were determined using a standard calculation [(mass of transgene DNA= (*N* bp transgene DNA × 1 µg genomic DNA)/3 × 10^9^ bp genomic DNA], assuming a haploid content of 3 × 10^9^ bp and hemizygosity of transgenic founder mice.

### SMA mice

‘Taiwanese’ SMA mice (*Smn*^−/−^*;SMN2^tg/tg^*) (35) purchased from Jackson Labs on a congenic FVB/N background were maintained following an established breeding strategy (36) and had a mean survival of 10/11 days (d). Litters were retrospectively genotyped using standard PCR protocols (35). Mice expressing the transgene (*SMN1*^SC^) were backcrossed to wild-type FVB/N mice (Harlan Laboratories) for 4 generations (93.75% homozygosity). A fifth generation backcross with FVB/N *Smn*^+/^^−^ mice generated pups with 96.87% homozygosity (*Smn*^+/−^;*SMN1*^SC^). A final backcross with ‘Taiwanese’ SMA mice (*Smn*^−/−^*;SMN2^tg/tg^*) generated four lines of experimental animals; control (*Smn*^+/−^; *SMN2*^tg/0^), control mice expressing *SMN1*^SC^ (*Smn*^+/−^; *SMN2*^tg/0^*;SMN1*^SC^), SMA mice (*Smn*^−/−^;*SMN2*^tg/0^), SMA mice expressing *SMN1*^SC^ (*Smn*^−/−^;*SMN2*^tg/0^;*SMN1*^SC^) (SMA rescue mice) (all >98.4% homozygosity). Litters from each backcross were genotyped at 14 d to determine *SMN1*^SC^ transmission. All mice were housed within the animal care facilities in Edinburgh under standard SPF conditions. All animal procedures and breeding were performed in accordance with Home Office and institutional guidelines.

### Immunohistochemical analysis of sectioned and teased nerve fibres

Sciatic nerve was fixed in 4% paraformaldehyde (PFA) for 30 min then washed in phosphate buffered saline (PBS). Prior to sectioning, nerves were embedded longitudinally in a 1:1 solution of 30% sucrose and OCT embedding matrix (CellPath). Ten micrometres of sections were prepared on slides. For nerve tease, the perineurium was carefully removed and fixed sciatic nerve fibres were teased in 0.1 Μ PBS on 3-amino propyltriethoxysilane (TESPA) coated slides. Slides were blocked for 0.5 h with 5% bovine serum albumin (BSA) and 0.2% Triton X-100. All nerves were incubated with primary antibodies raised against S100 (mouse, 1:100, Abcam) and FLAG (rabbit, 1:1000, ThermoFisher), then incubated in a solution of secondary antibodies (goat anti-mouse Alexa Fluor 555 and donkey anti-rabbit Alexa Fluor 488; both 1:500, Invitrogen). Slides were coverslipped using Mowiol (Calbiochem) or FluorShield (Abcam).

### Non-quantitative and quantitative fluorescent western blotting

Fresh tissue was dissected and frozen on dry ice and protein extracted in RIPA buffer (ThermoScientific) with protease inhibitor cocktail (Sigma). Non-quantitative western blots were performed using a primary antibody against SMN (mouse, 1:500; BD Transduction Laboratories). Odyssey secondary antibodies were added according to the manufacturers’ instructions (goat anti-mouse IRDye 680). Quantitative blots were carried out using a primary antibody against tubulin (mouse, 1:10 000; Abcam). Blots were imaged using an Odyssey Infrared Imaging System (Li-COR, Biosciences) at a resolution of 169 μM. Each blot was scanned and measured in triplicate to minimize user variability.

### Electron microscopy

Intercostal nerves were incubated for 48 h in 4% PFA: 2.5% glutaraldehyde at 4°C before post-fixation in 1% osmium tetroxide in 0.1 M phosphate buffer for 45 min. Following dehydration through an ascending series of ethanol solutions and propylene oxide, sections were embedded on glass slides in Durcupan resin. Regions to be used for the assessment of myelination were then cut out from a randomly selected section using a scalpel and glued onto a resin block for sectioning. Ultrathin sections (60 nm) were cut and collected on formvar-coated grids (Agar Scientific, UK), stained with uranyl acetate and lead citrate in an LKB Ultrostainer and then quantitatively assessed in a Philips CM12 transmission electron microscope equipped with a Gatan digital camera. Intercostal nerve fibres were measured using ImageJ. For each individual fibre, axon diameters and G-ratios were calculated as previously described (46).

### Assessment of SMA rescue mice

Righting reflex tests were performed to assess neuromuscular function, as previously described (37). Mice were weighed daily and Kaplan–Meier survival analyses performed as previously described (21). Muscle fibre diameter measurements were taken from phase-contrast micrographs of teased muscle fibre preparations using ImageJ software (47). NMJ pathology was assessed on whole-mount preparations of LAL muscles (39). Motor neuron cell body counts in the spinal cord were performed as previously described (40,48).

### Microscopy

Fluorescent images were captured using a Zeiss 710 laser-scanning confocal microscope (40× objective; 1.4NA) or a standard epi-fluorescence microscope equipped with a chilled CCD camera (20× or 40× objective; 0.8NA; Nikon IX71 microscope; Hammamatsu C4742-95), as previously described (49).

### Statistical analysis

All data were collected into Microsoft Excel and analysed using GraphPad Prism software (statistical tests used for each comparison are detailed in the text). For all statistical analyses, *P* < 0.05 was considered statistically significant. All data are expressed as mean ± SEM.
